# Injectable Poly-l-Lactic Acid for Body Aesthetic Treatments: An International Consensus on Evidence Assessment and Practical Recommendations

**DOI:** 10.1007/s00266-024-04499-9

**Published:** 2024-11-26

**Authors:** Alessandra Haddad, Luiz Avelar, Sabrina G. Fabi, Juliana Sarubi, Michael Somenek, Daniel Dal’Asta Coimbra, Melanie Palm, Kalpna K. Durairaj, Munir Somji, Roberta Vasconcelos-Berg, Lim Hanseok, Kate Morlet-Brown, Jeff Huang, Rebecca Fitzgerald, Doris Hexsel, Cheri Mao, Felipe Weinberg, Inna Prygova, Daniel Bråsäter

**Affiliations:** 1 Hospital Israelita Albert Einstein, Federal University of São Paulo, Rua Bandeira Paulista 726, 8th Floor, São Paulo, SP 04532-002 Brazil; 2 A Haddad Medical and Aesthetic Clinics, UNIFESP Federal University of São Paulo, São Paulo, Brazil; 3 Clinica Luiz Avelar, Belo Horizonte, Brazil; 4 Cosmetic Laser Dermatology, San Diego, CA USA; 5 Dermatology Private Practice, Belo Horizonte, MG Brazil; 6 Advanced Plastic Surgery, Washington, DC USA; 7 Instituto de Dermatologia Professor Rubem, David Azulay Santa Casa de Misericordia/IDPRDA, Rio de Janeiro, Brazil; 8 Art of Skin MD, Solana Beach, CA USA; 9 Beauty By DrKay, A Medical Corporation, Pasadena, CA USA; 10 DrMediSpa, London, UK; 11 University Hospital of Basel, Basel, Switzerland; 12 Yonsei Medinoble Clinic, Seoul, Korea; 13 Waikiki Specialist Centre, Waikiki Private Hospital, Waikiki, WA Australia; 14 Taipei, Taiwan; 15 Los Angeles, CA USA; 16 Hexsel Dermatologic Clinics and Brazilian Center for Studies in Dermatology, Porto Alegre, Brazil; 17 Galderma, Princeton, NJ USA; 18 Galderma, Dallas, TX USA; 19 Galderma, Uppsala, Sweden

**Keywords:** Poly-l-lactic acid, PLLA-SCA, Biostimulator, Body aesthetics, Body rejuvenation

## Abstract

Non-facial (body) rejuvenation is a treatment of increasing demand, with patients seeking to harmonize the benefits of rejuvenated facial appearance with other areas of the body. Poly-l-lactic acid (PLLA)-SCA (Sculptra®) has been approved for facial aesthetic uses since 1999 in Europe and since 2009 in the USA and more recently evaluated for the treatment of cellulite of the buttocks and thighs and other body indications. The current evidence base consists largely of prospective observational analyses and case series although systematic evaluations for a number of body areas are underway. Current data support a potential benefit for the use of PLLA-SCA for the aesthetic treatment of the neck, knees, abdomen, hands and upper arms. Improvements in aesthetic appearance (e.g. sagging, wrinkling, dimpling, cellulite) that are sustained over time with minimal side effects can be achieved. Standardization of injection protocols for different body areas is now needed along with the validation of clinical tools that can be used to agree on treatment goals and to evaluate aesthetic improvements over time. A group of international experts in the fields of facial and body aesthetics, plastic surgery and dermatology were selected based on their educational, scientific and publication merits together with clinical experience using PLLA-SCA for body rejuvenation. Here, we provide an evidence-based and expert-led consensus (14 years of off-face treatment experience) on recommendations for appropriate injection protocols for different body sites and evaluation tools when using the biostimulator PLLA-SCA for body aesthetic and rejuvenation procedures.

*Level of Evidence IV* This journal requires that authors assign a level of evidence to each article. For a full description of these Evidence-Based Medicine ratings, please refer to the Table of Contents or the online Instructions to Authors www.springer.com/00266.

## Introduction

The number of people seeking non-surgical aesthetic treatment is increasing around the world. A global survey conducted by the International Society of Aesthetic Plastic Surgery [1] reported that more than 18.8 million non-surgical aesthetic procedures were performed in 2022. Injectables accounted for 13.8 million of these procedures, a 39.4% increase since 2018 [1].

Non-facial (body) rejuvenation is a treatment of increasing demand with patients seeking to harmonize the benefits of a rejuvenated facial appearance with other areas of the body. Areas of interest include the neck and décolletage, buttocks, thighs, abdomen, hands, knees and upper arms. Women over 40 years of age remain the main consumers of aesthetic treatments, seeking to regain a more youthful appearance [2]. However, men and younger people wishing to delay the skin and body shape changes that signal aging are increasingly seeking non-invasive, natural looking aesthetic treatments [1, 3–5]. Patients are also seeking minimally invasive treatments that offer improved skin appearance in addition to a contouring effect to address issues such as laxity and cellulite-related dimpling. An additional emerging use for poly-l-lactic acid is in correcting post-surgical soft tissue deformities and post-partum abdominal laxity [6].

The use of injectables for body rejuvenation has been largely driven by requests from patients. However, larger studies or clinical trials that evaluate the injection protocols, efficacy, durability and patient's satisfaction with those treatments have been lacking. Experience has been shared in the form of case series, practitioner-led investigations and expert consensus panels. Poly-l-lactic acid (PLLA)-SCA (Sculptra®; Galderma, Sweden) is an injectable, biodegradable biostimulator that promotes the production of collagen and elastin by stimulation of regenerative pathways [7–11]. PLLA-SCA has been approved for facial aesthetic uses since 1999 in Europe and since 2009 in the USA. Most recently, in a prospective, single-centre, double-blind, split-body study in women with slight to moderate skin laxity of the buttocks and/or thighs (NCT04830722), PLLA-SCA resulted in a significant reduction of depression depth and improvement of the appearance of the skin in both areas with no significant side effects reported [12]. Additional studies have been completed and are in the process of being published, evaluating PLLA-SCA for the treatment of arm laxity (NCT05445661), décolletage wrinkles (NCT05538728), hip dell profile (NCT05269654) and cellulite in the thighs (NCT05064761), together with a multicentre retrospective chart review that evaluated the safety of PLLA-SCA when used in non-facial areas (NCT05463978). Other PLLA-based injectables are available in a limited number of countries. However, differences in molecular shapes, sizes and hydrolytic degradation [13] of the PLLA molecules and the excipients used in the formulations may confer fundamental differences in safety and performance, including stimulation of collagen production [14], although head-to-head evaluations are lacking.

This review presents an international expert consensus on the current use of the biostimulator PLLA-SCA for body aesthetic and rejuvenation procedures, emerging insights and recommendations for appropriate injection protocols for different body sites and evaluation tools, as well as areas for future research.

## Objective

To provide an evidence-based and expert-led consensus on recommendations for appropriate injection protocols for different body sites and evaluation tools when using the biostimulator PLLA-SCA for body aesthetic and rejuvenation procedures.

## Methods

International experts in the field of facial and body aesthetics, plastic surgery and dermatology were selected based on their educational, scientific and publication merits together with clinical experience using PLLA-SCA for body rejuvenation and willingness to participate in the project. Additionally, the experts selected had a broad geographical reach to provide a global view in order to capture any differences in use of poly-l-lactic acid geographically occurring because of differences in ethnicity, diversity and patient profiles.

Each expert panel member collated all published articles on the non-facial use of PLLA-SCA of which they were aware. These were then compiled, any duplicates removed, and organized by treatment area (neck and décolletage, buttocks, knees, abdomen, hands and upper arms). The experts then discussed each publication and selected those identified as being of relevance to the objectives of the article. Where possible, the experts also identified any areas of concern and possible future directions.

### Biostimulation with PLLA-SCA for Body Indications

PLLA has been used for decades for a variety of clinical applications including medical implants, suture materials and as part of dissolvable meshes for orthopaedic use [15]. In the early 2000s, PLLA-SCA was approved for the aesthetic treatment of human immunodeficiency virus (HIV)-related facial lipoatrophy and subsequently for facial contour deficiencies [16]. Studies for these indications demonstrated the effective and long-lasting volumization that could be achieved with subdermal injections of PLLA-SCA [16]. The more recent recognition of the effect of PLLA-SCA on collagen and elastin production via stimulation of regenerative pathways [7, 10] has added another dimension to its potential in aesthetic regenerative medicine [8–11, 17]. Collagen production is stimulated by the presence of PLLA-SCA microspheres through the body’s natural foreign body response [14]. This involves a mild and localized inflammatory response that persists for several months as immune cells including lymphocytes and macrophages migrate to the site and coat the PLLA-SCA microspheres. In the months following injection, the PLLA molecules are gradually degraded to carbon dioxide and water and collagen is deposited in the extracellular matrix, restoring and maintaining tissue volume and soft-tissue support [18]. To achieve optimal outcomes for patients, an understanding of the structural components of the skin and underlying musculature is essential in order to develop safe and effective injection protocols that will achieve the required volumizing effect and effectively address skin quality issues such as laxity, cellulite and integrity (dermal thickness, foundation and aging changes).

### Neck and Décolletage

Minimally invasive treatments to address skin laxity and wrinkles in the neck and décolletage area are highly desirable in this aesthetically important area. In addition to age-related skin wrinkling, these areas are at increased risk for photodamage leading to skin alterations such as fine wrinkles, laxity, dermal thinning, and colour changes [19].

An open, prospective study in 25 healthy females with moderate-to-severe crepiness and wrinkling of the décolletage demonstrated the benefit and safety of PLLA injection to address these issues with sustained improvements in a 6-month study [20]. Subjects were injected with 1 vial containing 150 mg of PLLA-SCA diluted with 9 mL of sterile water at each of three treatments using a 27-gauge needle. Outcomes were assessed using the Fabi-Bolton 5-point chest wrinkling scale (1 = wrinkles absent; 2 = shallow but visible wrinkles; 3 = moderately deep wrinkles; 4 = deep wrinkles, with well-defined edges; 5 = wrinkles very deep with redundant folds) at baseline and at 1, 3 and 6 months after treatment. All post-treatment scores were statistically significantly improved compared with baseline by both investigators and subjects. At least one-point improvements were reported at Month 1 by 83% of investigators and 74% of subjects. At Month 6, 90% of investigators and 57% of subjects still reported improvement in appearance of wrinkles from baseline. No adverse events were reported at any time during the study. A number of retrospective case series have also examined the use of PLLA-SCA for the neck and décolletage areas [21–23]. Mazzuco & Hexsel [22] reported a case series of 36 subjects who received PLLA-SCA to the neck and chest diluted with 10 mL of sterile water with added 2% lidocaine (0.1 mL to each 0.9 mL of solution) using a 27-gauge needle. The focus of the evaluation in these patients was aesthetic improvement evaluated using the Global Aesthetic Improvement Scale (GAIS) and subject satisfaction. Using before and after photographs of 21 subjects taken 60 days after the last treatment, three independent evaluators reported visible alterations with improvement ranging from 81% to 100%. In addition, 92% of patients reported being pleased with the result of treatment and stated that they would undergo treatment again. The authors reported that the results were maintained at 18 months after treatment. One case of early onset (within 1 week) multiple nodule formation in the anterior neck region was reported by one patient. Nodules were only visible upon hyperextension. The nodules were treated with an injection of sterile water and vigorous massage, with the patient instructed to massage the area 3 times daily for 10 days. At the 60-day follow-up evaluation, around 80% of the nodules had regressed and the remaining nodules were treated with intralesional triamcinolone. Redaelli & Forte [23] reported a retrospective review of 568 patients treated with PLLA-SCA for various body areas including the neck for 162 subjects and décolletage for 72 subjects between 1999 and 2007. The authors used a dilution volume of 5–8 mL for each 150 mg PLLA-SCA vial. Subjects were followed up every 3 months for the first year after their final treatment. Across all patients, the average Definitive Graduated Score for satisfaction (1: least satisfied; 10: most satisfied) was 7.8 (range 6.3–8.4). The authors reported that the improvement in skin appearance and lifting effect (tightening the skin's collagen fibers to help skin resist the downward force of gravity and look more toned) persisted for a period of months after treatment. The principal side effect was late onset non-infectious nodules which occurred in 1% of subjects, 4 of which were described as cold (non-inflammatory) nodules and 2 were described as warm (inflammatory) nodules. In a retrospective review of 28 cases in which PLLA was used for the aesthetic treatment of the décolletage area, improvements of 1 to 2 points from baseline on the Fabi-Bolton 5-point chest wrinkling scale were recorded for 28 females (mean age 52.9 years) [21]. Subjects received a mean of 2.3 treatments (range 1–7) with at least 4 weeks between each treatment. The authors reported that the best improvements were noted for patients who received at least 3 PLLA-SCA injection sessions at a 16 mL dilution with 16 mL injected per treatment.

**Fig. 1:**
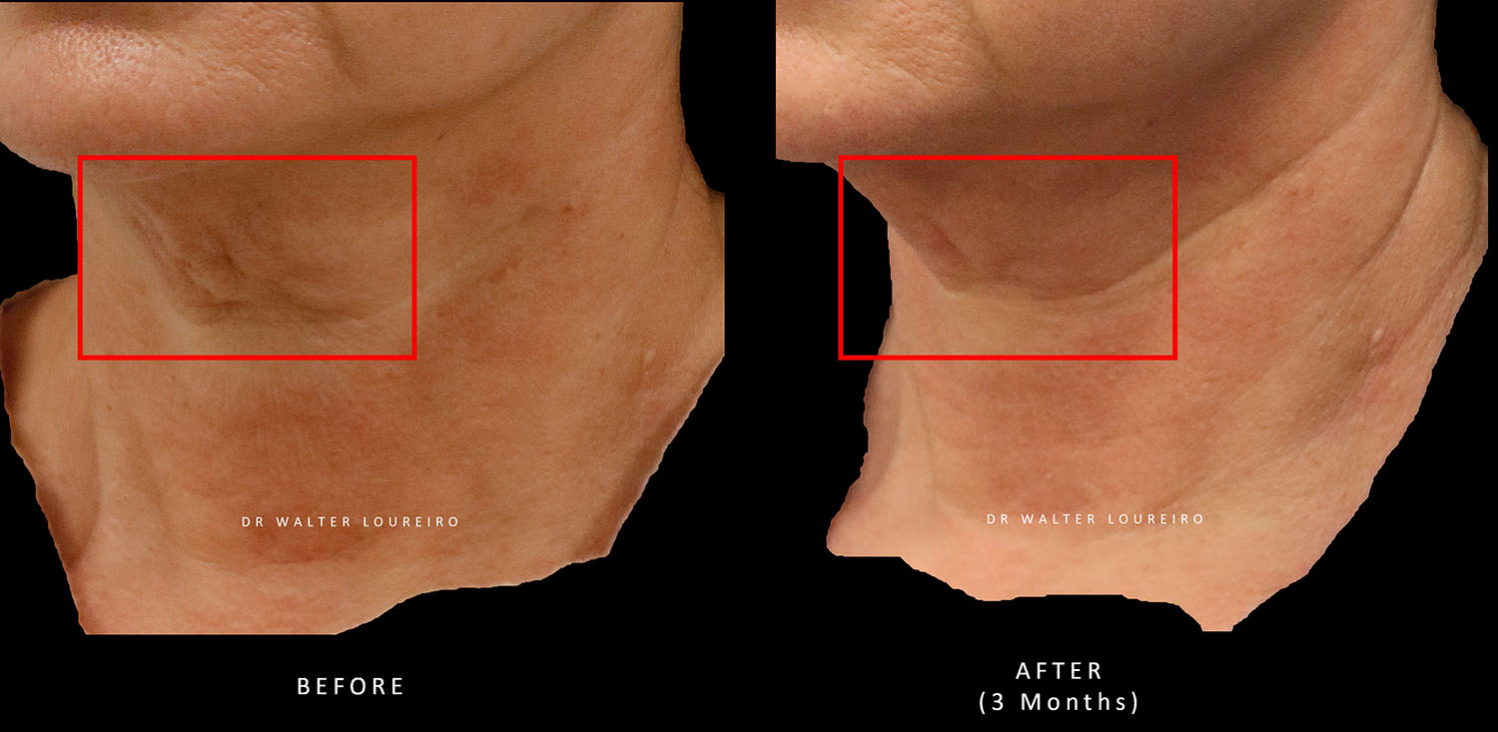
Before and after treatment with PLLA-SCA in the neck region using 1 vial of PLLA-SCA. Pictures courtesy of Dr Walter Loureiro

**Fig. 2:**
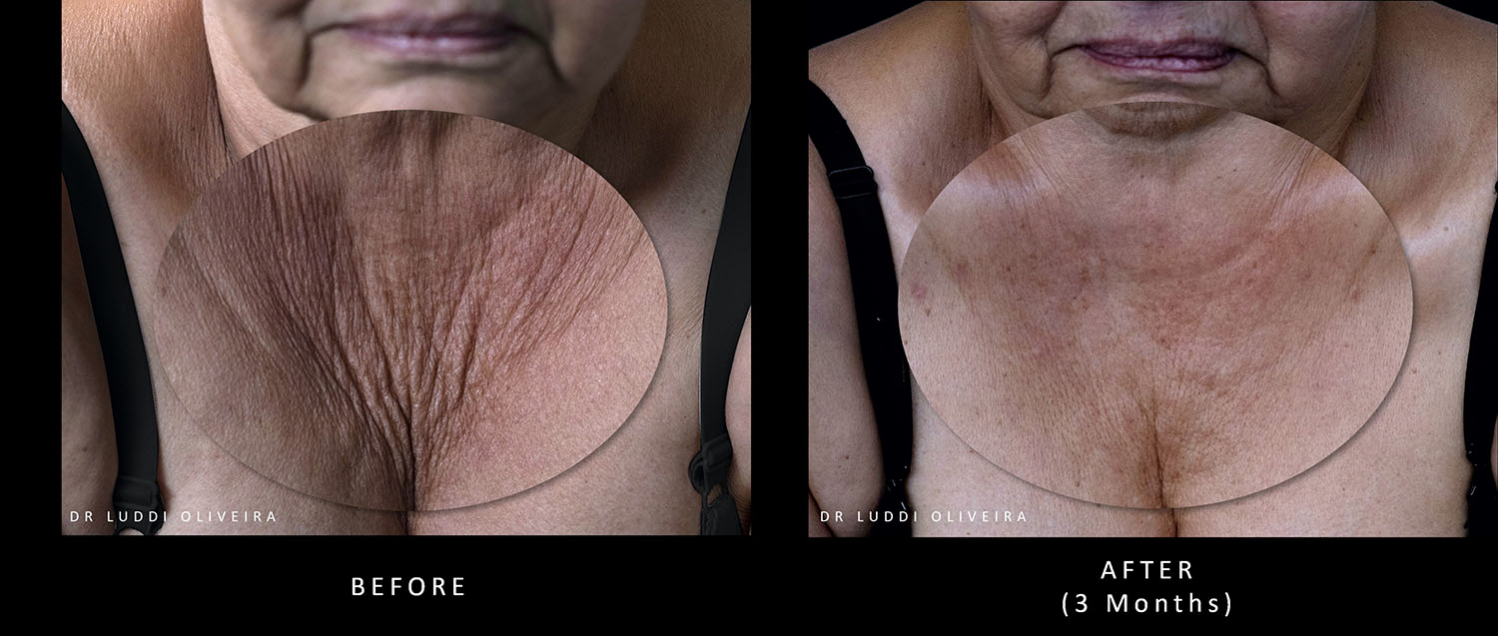
Before and after treatment with PLLA-SCA in the décolletage area using 2 vials of PLLA-SCA. Pictures courtesy of Dr Luddi Oliveira

Images of patients’ neck and décolletage regions before and after treatment with PLLA-SCA are shown in Figs. 1 and 2, respectively.

#### Expert Consensus

The evidence base supports a potential benefit for the use of PLLA-SCA for the aesthetic treatment of the neck and décolletage. Dilution volumes of 8–17 mL should be considered with the addition of lidocaine and injected using either a blunt cannula or a 25-gauge needle (Table 1). At least 2–4 treatment sessions should be considered with an interval between sessions of at least 4 weeks. Post-treatment massage of the injected area (vigorous massage using liquid soap immediately after treatment) is recommended to reduce the risk of nodule formation. There is a need for a clinical study of the proposed injection protocol with evaluation of aesthetic improvement (e.g. GAIS), improvement of crepiness/wrinkles (e.g. Fabi-Bolton 5-point chest wrinkling scale) and subject satisfaction (satisfaction with treatment questionnaire). Evaluation of muscular hyperactivity and volume alterations should be considered before treating neck laxity to optimize treatment outcome.

**Table 1:** Expert recommendations for the use of PLLA-SCA for selected body aesthetic treatments

Region	Recommendations for the use of PLLA-SCA
Neck/chest/décolletage	Total dilution volume*: 8 or 17 mL Include lidocaine: Yes Needle/cannula: 25–26-gauge needle or blunt cannula Injection protocol: At least 2–4 sessions at 4-week intervals. Post-treatment massage of injected areas (to reduce risk of nodule formation)
Gluteal	Total dilution volume*: 17 mL Include lidocaine: Yes Needle/cannula: 25–26-gauge needle or 22–25-gauge stiff cannula Injection protocol: At least 3 sessions at 4–6-week intervals. Deeper injection for lifting effect in upper quadrant only (70% of product)
Knees	Total dilution volume*: 17 mL Include lidocaine: Yes Needle/cannula: 25–26-gauge needle or 22-gauge cannula Injection protocol: Extending the treatment area to superior to the knee joint might have additional benefit
Abdomen	Total dilution volume*: 17 mL Include lidocaine: Yes Needle/cannula: 25-gauge needle Injection protocol: Up to 3 sessions with a 45-day interval. Always include the area superior to the umbilicus (especially for a lifting effect)
Hands	Total dilution volume*: 9 mL Include lidocaine: Yes Needle/cannula: 25-gauge needle Injection protocol: Additional studies required; avoiding joints, lifting skin away from underlying tendons, using high-volume dilution with thin skin might have more beneficial outcomes
Upper arms	Total dilution volume*: 17 mL Include lidocaine: Yes Needle/cannula: 25-gauge needle or 22-gauge cannula Injection protocol: 1 vial per side per session, always treat the inner and medial region. In moderate-to-severe cases, 1.5 vials per side, treat the inner, medial and posterior arms for 2–4 sessions with a minimum 30-day interval

*Per 150 mg vial of PLLA-SCA

### Buttocks

The effect of age-related volume and contour loss in the gluteal region can result in a sagging of the buttocks, increased appearance of cellulite, more pronounced ‘hip dip’ and flattening of the gluteal fold. Aesthetic treatments for the gluteal region may include volumization and/or contouring to reduce the appearance of ‘hip dip’ and improve the buttock silhouette, elevate tissue above the gluteal fold, improvement in skin texture and smoothing of dimpling.

PLLA-SCA has been evaluated for the treatment of cellulite of the buttocks and thighs in a prospective, single-centre, double-blind, split body study [12]. The study included 20 women with slight to moderate skin laxity of the buttocks and/or thighs who received injections with up to 2 vials of PLLA-SCA (16 mL dilution) or a control injection (bacteriostatic water) per treatment area for a total of 3 treatments, 4 weeks apart. A fanning technique in the subdermal-high subcutaneous layer by needle or cannula was used to perform the injections. Treatment with PLLA-SCA resulted in a 40% reduction in the Hexsel CSS depression depth score and a 22.5% improvement in the Hexsel CSS morphological appearance of the skin score in the buttock region with no significant side effects reported. A prospective, multicentre, single cohort, open-label trial investigated the efficacy and safety of PLLA-SCA for the aesthetic treatment of gluteal contour deformities [24]. The study included 30 female subjects who received bilateral PLLA-SCA injections over 3 treatment sessions with 4 weeks between each session and were followed for up to 6 months. The reconstitution volume was 16 mL with an additional 2 mL volume of 1% lidocaine added just prior to injection, administered via a 22-gauge cannula into the deep dermis/subcutaneous regions using threading, fanning or tunnelling technique. Up to 3 vials could be used per buttock (up to 6 vials per session) with injections spaced at a distance of 0.5–1 cm apart over an approximate area of 16 cm x 20 cm per buttock. The authors used different injection protocols depending on the desired effect for individual patients. For example, deeper injections in the upper lateral quadrant were used to achieve a lifting effect. More superficial injection was used to address skin dimpling. At 6 months after the final treatment >80% of subjects continued to display a 1-point improvement on the GAIS, the primary endpoint of the study. Improvements in skin hydration, elasticity, roughness and scaliness were also reported. The most commonly reported adverse events were injection-related (bruising, swelling, redness, pain/tenderness) and were of short duration, resolving within days. No serious adverse events were reported. A previous open-label study in 14 females [25] also reported improved appearance of the gluteal area through the 6 months of study follow-up. In this smaller study, subjects were injected with 1 vial (150 mg) of PLLA-SCA per session (across both buttocks) diluted to an 8 mL volume with an additional 2 mL lidocaine. Treatment was delivered at 2 sessions with a 45-day interval between sessions. After 6 months, 85% of subjects and 100% of investigators reported an improvement in the general appearance of the skin in the gluteal area. The study also included an ultrasound evaluation of dermal thickness 6 months after the initial treatment and reported an increase in thickness in 11 of the 14 subjects. In 2021, Swearingen and colleagues [26] reported the results of a randomized, placebo-controlled study in 31 females with lower extremity (gluteal and thigh) cellulite. Each 150 mg PLLA-SCA vial was diluted to 10 mL with an additional 2 mL lidocaine and half to one vial was delivered on each side of the buttock or thigh using a 26-gauge needle. At the 3- and 6-month follow-up, statistically significant improvements were noted for PLLA-SCA compared with placebo using the GAIS compared to baseline as assessed by blinded investigators. A retrospective clinical review of 60 subjects who underwent treatment with PLLA-SCA for gluteal augmentation also reported visible volumization, improved skin texture and a reduction in cellulite dimpling (using the GAIS scale) over 2 years following treatment [27]. Subjects received between 1 and 3 treatment sessions 4–6 weeks apart and were treated with between 2 and 12 vials per session, depending on patient budget. When patients were treated with at least 20 vials, Durairaj and colleagues [27] found that PLLA-SCA allows for visible volume amplification, improved skin texture, and softened cellulite dimpling in the buttocks (using the GAIS scale). No adverse events were reported, and the treatment was found to be safe and effective for gluteal augmentation. A further case series of 20 subjects treated with PLLA-SCA for volumization, cellulite and skin quality improvement in the buttocks, thighs and abdomen, mainly for post-surgical soft tissue deformities, has also been reported [6]. Improvements were noted following 86% of treatment sessions with transitory mild, injection-related adverse events reported. Nodule formation was reported in 1 patient, was considered mild in severity, and resolved spontaneously after 38 days.

Sarubi and colleagues [28] describe a targeted and individualized technique of PLLA-SCA injection into the buttock area that involves clinical and anatomical evaluations of the gluteal region; there are three distinct approaches for injecting PLLA in the gluteal region based on the most important factor to be improved: (1) skin quality, (2) contour and lifting, or (3) projection and volume [28]. This technique was associated with favourable patient outcomes in terms of improvements in skin quality and laxity, contour and lifting, and gluteal volume and projection, all of which were achieved with a lower volume of PLLA compared with other PLLA injection techniques.

#### Expert Consensus

Expert recommendations for the use of PLLA-SCA for the aesthetic treatment of the gluteal area have recently been issued [29]. Dilution volumes of 17 mL should be considered with the addition of lidocaine and injected using either a 25-gauge needle or a 20–22-gauge stiff cannula. Up to 3 treatment sessions should be considered with a 4-to-6-week interval between sessions. Given the range of possible aesthetic effects that can be achieved, subject goals of treatment should be carefully evaluated prior to treatment as this will inform the location, spread and depth of injection. The recent development of a simple clinical tool, the Buttock Assessment Scale, can be considered to assist with determining subject goals of treatment and evaluating improvement over time [30]. The scale enables identification, scoring and evaluation over time of a range of gluteal aesthetic issues including contour, projection, volume loss, skin laxity, infragluteal fold shape, skin integrity concerns (such as the presence of stretch marks, hollows or blemishes) and cellulite.

**Fig. 3:**
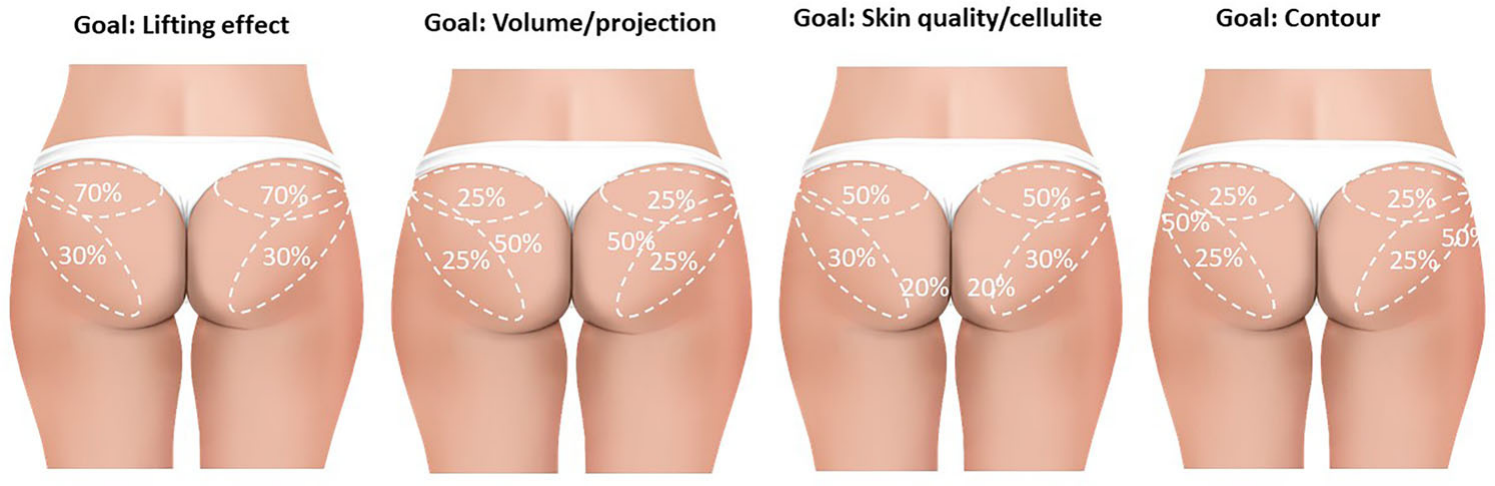
Suggested target areas and distribution of PLLA-SCA injection for gluteal aesthetic treatments

The anatomical structure of the gluteal region is of critical importance in determining the optimal positioning of PLLA-SCA to achieve the desired aesthetic effects [31, 32]. Superficial subcutaneous injection above the superficial fascia in the upper quadrant of the buttocks is suitable for a lifting effect and reduction of the appearance of the gluteal fold (Fig. 3). A product distribution of 70% in the upper region of the buttocks with 30% in the lower region would be an appropriate distribution [28]. The group acknowledged that consensus was required as to whether additional vials/session or multiple sessions would provide better outcomes for patients.

### Knees

The efficacy and safety of PLLA-SCA for the treatment of skin laxity and wrinkles in the knee area has been evaluated in a single randomized controlled trial [33]. A total of 20 female subjects received three sessions of PLLA-SCA (each 150 mg vial diluted to 14 mL with the addition of a further 2 mL 1% lidocaine) in one knee and the same volume of bacteriostatic water to the other knee. Subjects were treated with 1 vial/knee over 3 treatment sessions, each separated by 4 weeks. The authors reported statistically significant improvements in skin laxity for the PLLA-SCA-treated knee vs the placebo-treated knee as assessed using the physician GAIS 28 days after treatment, with improvements sustained at 84 days and 168 days. PLLA-SCA treatments were well tolerated without any significant adverse events. There were no cases of oedema, contour irregularity, or nodules in either the active- or placebo-treated knees [33].

**Fig. 4:**
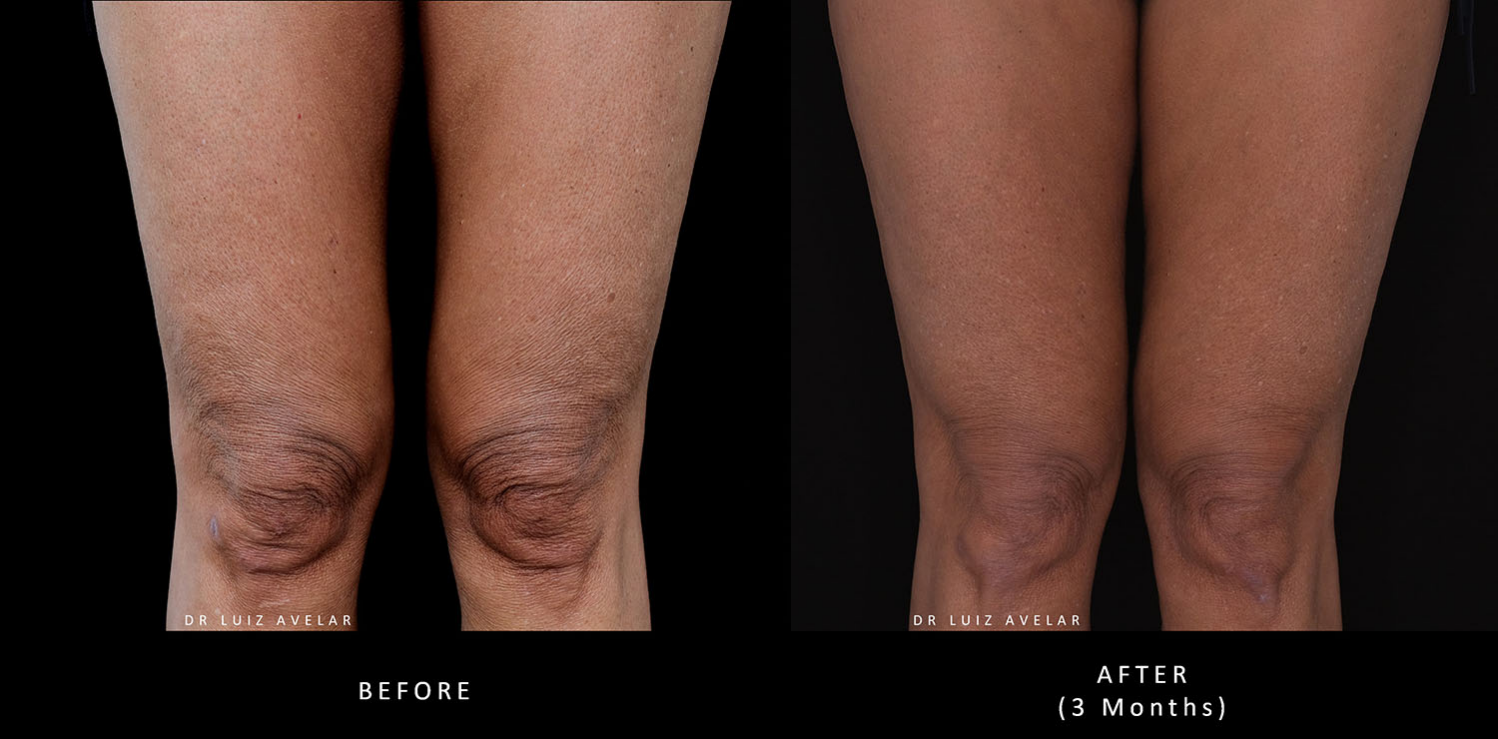
Before and after treatment with PLLA-SCA in the knee area using 0.5 vial of PLLA-SCA per side. Pictures courtesy of Dr Luiz Avelar

Images of a patient’s knees before and after treatment with PLLA-SCA are shown in Fig. 4.

#### Expert Consensus

Data suggest a potential benefit for PLLA-SCA for the treatment of skin laxity of the knee. A total dilution volume of 16 mL (to include 1 mL lidocaine) for a 150 mg vial of PLLA-SCA is recommended per knee injected using a 25-gauge needle or 22-gauge cannula. Extending the treatment area to superior to the knee joint might have additional benefit. Additional studies are required to determine the most appropriate dilution volume and injection protocol, as is a validated tool for the evaluation of change in skin laxity and wrinkling in this area.

### Abdomen

Abdominal skin laxity can emerge due to aging and following pregnancy or significant weight loss. While moderate-to-severe skin laxity in this area may require surgical intervention, a non-invasive approach may be suitable for people with mild skin laxity or those who do not wish to undertake a major surgical intervention. To date only a single case series has been reported for the use of PLLA-SCA for abdominal aesthetic treatments [34]. In 2017, Sadick & Arruda [34] reported the use of PLLA-SCA for abdominal treatment of 5 subjects to improve skin quality, texture and to enhance the contours of the area. The dilution volume was 8 mL with an additional 2 mL of lidocaine and 1 to 2 vials injected per treatment session using a 27-gauge needle. Patients were treated with 1–2 vials in 1–2 sessions with sustained marked improvement at the 12-month assessment. Only mild bruising at the injection sites was reported, which spontaneously resolved after 3 weeks.

**Fig. 5:**
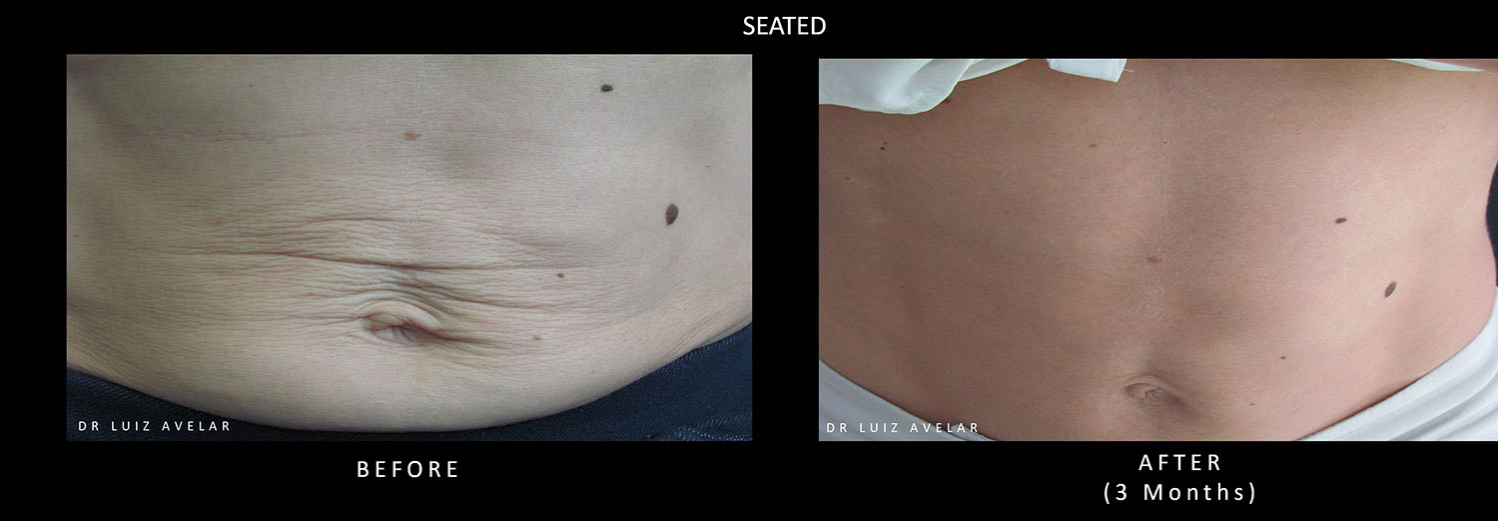
Before and after treatment with PLLA-SCA in the abdominal region using 3 vials of PLLA-SCA. Pictures courtesy of Dr Luiz Avelar

Images of a patient’s abdomen before and after treatment with PLLA-SCA are shown in Fig. 5.

#### Expert Consensus

For treatment of the abdominal area a total dilution volume of 17 mL (to include 1 mL lidocaine) for a 150 mg vial of PLLA-SCA is recommended, using 1 to 3 vials per session according to the severity of the laxity. Up to 3 sessions can be necessary with a 45-day interval. It is important to always include the area superior to the umbilicus in the treatment, especially the central abdominal area, located over the rectus abdominis muscles. For more of a lifting effect, injection areas superior to the umbilicus are important. Additional studies are required to determine the most appropriate dilution volume and subcutaneous injection protocol for the treatment of abdominal skin laxity. A validated tool for the evaluation of change in skin laxity and wrinkling in this area is also needed. The management of patient expectations and understanding of the extent of abdominal skin laxity that can be effectively managed with a non-invasive approach using PLLA-SCA should be explored. The group noted that as patients will often receive additional treatments such as ultrasound or muscle stimulation in this area, studies are needed to define the contribution of all procedures on outcomes.

### Hands

Injectable biostimulators have been proposed as a potential option for the treatment of soft tissue atrophy of the hand including thinning of the intermetacarpal spaces [23, 35–37]. The safety of PLLA-SCA for the treatment of volume loss of the hands has been reported for a series of 26 subjects treated at 3 clinical practices in the USA [38]. The dilution volume was 8–10 mL including lidocaine per vial across the 3 clinical centres, with 1–3 vials injected into both hands per treatment session for 2–3 sessions using a 25-gauge needle. The majority of adverse events reported were related to the injection procedure (bruising, swelling and pain) and resolved within days of treatment. There were no reported cases of papules or nodules and no serious adverse events reported. This retrospective evaluation also found that the majority of subjects were very satisfied with the improvement in volume achieved. A further retrospective evaluation included 8 patients who received PLLA-SCA for hand volume loss [39]. The average dilution volume was 10.25 mL (including 1 mL of lidocaine 1%), injected into the upper cutaneous compartment using a 25–26-gauge needle and using one vial per treatment session. In all, 63% of patients reported good to excellent correction for hand volume loss. In this study, a non-inflammatory nodule formation was reported in 1 of 8 cases (12.5%), after an injection of one vial of PLLA-SCA 150 mg diluted in 9 mL. The total amount used per hand was not described. Deep dermal injection has also been used to add volume to intermetacarpal spaces [23, 40].

**Fig. 6:**
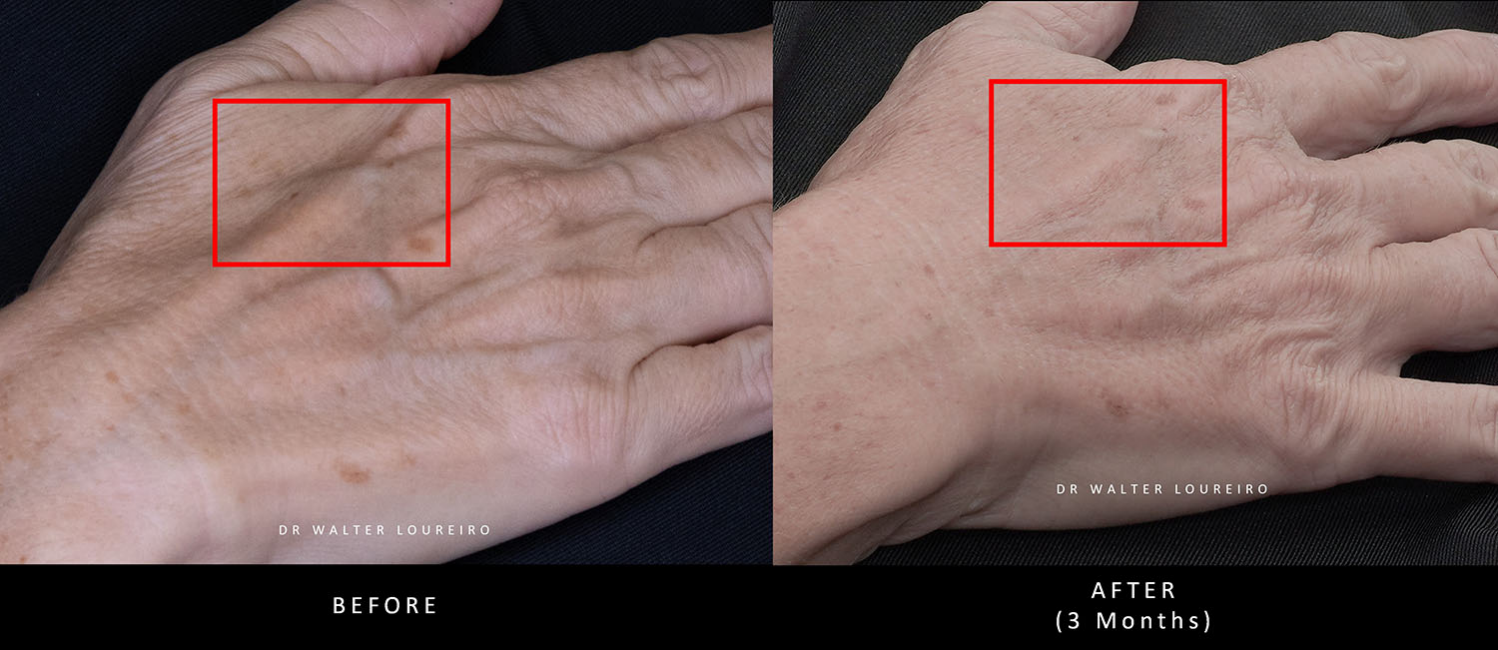
Before and after treatment with PLLA-SCA in the hands using 0.5 vial of PLLA-SCA per side. Pictures courtesy of Dr Walter Loureiro

Images of a patient’s hands before and after treatment with PLLA-SCA are shown in Fig. 6.

#### Expert Consensus

For the dorsal hand, a total dilution volume of 9 mL (to include 1 mL lidocaine) for a 150 mg vial of PLLA-SCA is recommended. Additional studies are required to determine the most appropriate dilution volume and subcutaneous injection protocol for the treatment of volume loss, skin thinning and wrinkling of the hand with PLLA-SCA. Avoiding the joints, lifting skin away from underlying tendons and using a high-volume dilution with thin skin might have more beneficial outcomes. In addition, protocols for post-injection care (e.g. massage) to minimise nodule formation would be of benefit.

### Upper Arms

A small number of cases in which subjects received PLLA-SCA for the aesthetic treatment of laxity in the upper arm have been reported [6, 23, 41, 42]. Dilution volumes of 10–20 mL and both deep dermal and superficial injection protocols were used with needle and cannula. Dal’Asta Coimbra & Amorin [41] used a dilution volume of 20 mL (16 mL of distilled water and 4 mL of 2% lidocaine) and, having divided the region of the arm to be treated into four quadrants, PLLA-SCA was applied using the linear retrograde technique, injecting into the deep dermis in parallel cylinders. Approximately 1.25 mL of PLLA-SCA was used in each quadrant, totalling 5 mL per arm. After application, vigorous massage was performed on the treated area for 10 minutes, and patients were instructed to use the same massage technique twice daily for 10 days. The number of sessions ranged from 2–4, at intervals of approximately 4 weeks. Four weeks after the first application, there were improvements in skin texture of the treated area and subject satisfaction with outcomes, and there were reductions in sagging and in cellulite. These results were even more evident after the second application and improvements remained after 22 months. Side effects of pain during the application, local erythema, and transient hematoma were reported but there were no reports of nodule formation [41].

**Fig. 7:**
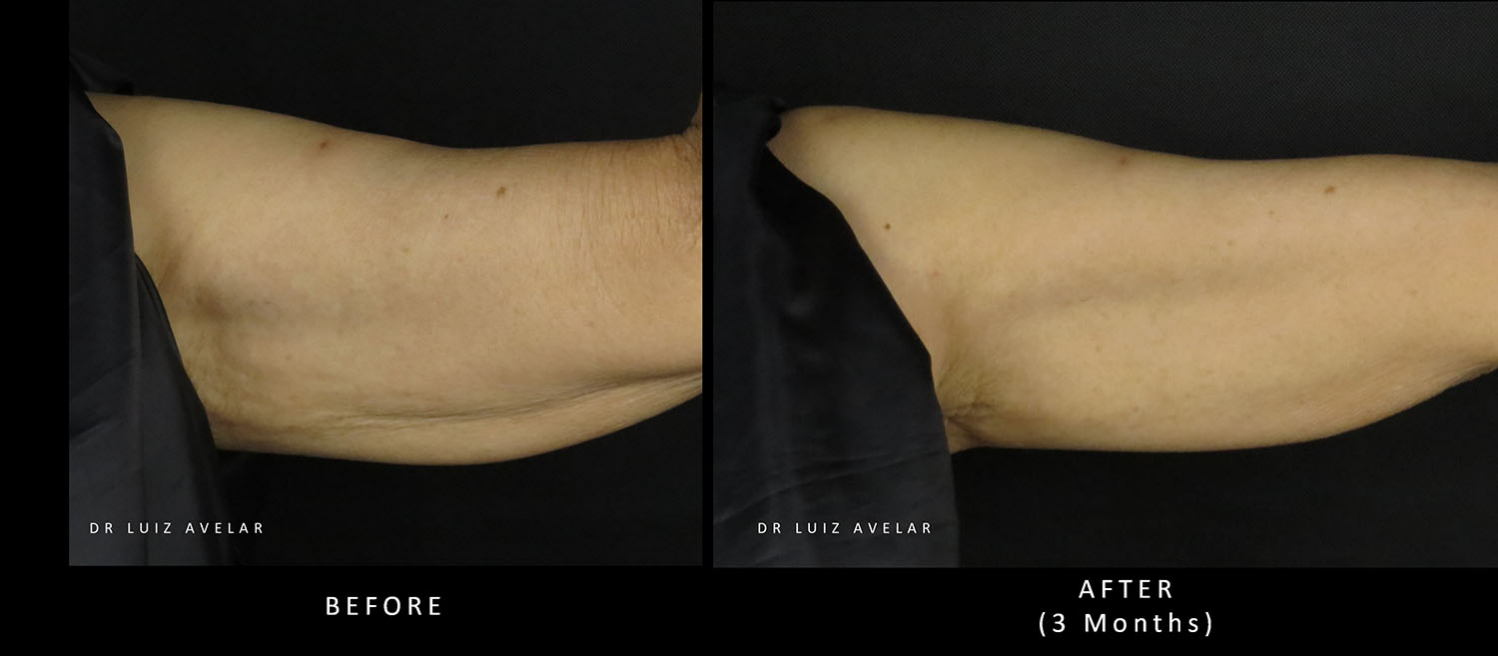
Before and after treatment with PLLA-SCA in the upper arm using 3 vials of PLLA-SCA over 3 sessions. Pictures courtesy of Dr Luiz Avelar

**Fig. 8:**
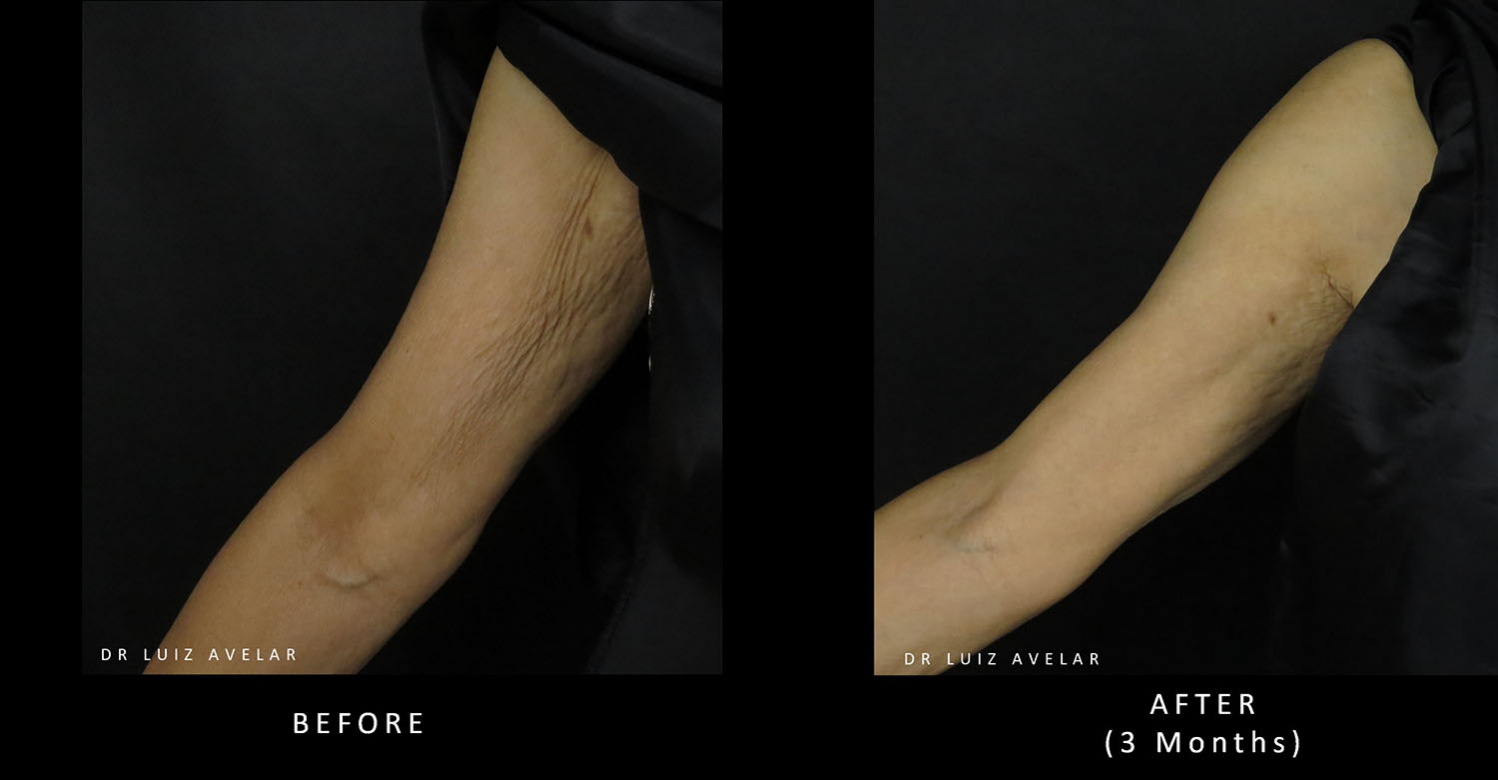
Before and after treatment with PLLA-SCA in the upper arm using 3 vials of PLLA-SCA over 3 sessions. Pictures courtesy of Dr Luiz Avelar

Two cases showing images of patients’ upper arms before and after treatment with PLLA-SCA are shown in Figs. 7 and 8.

#### Expert Consensus

PLLA-SCA has the potential to address aesthetic concerns in the upper arms including skin laxity and wrinkling. Visible improvement has been achieved with both deep dermal and superficial subcutaneous injection. Additional studies are required to determine the most appropriate dilution volume and injection protocol for the aesthetic treatment of the upper arm with PLLA-SCA. Initial recommendations would be to use a dilution volume of 17 mL using a 25–26-gauge needle or 22-gauge cannula, injecting on a subcutaneous plane using 1 vial per side per session. In moderate-to-severe cases, 1.5 vials of PLLA-SCA per side are recommended for 2–4 sessions with a minimum interval of 30 days. The inner, medial and posterior arms should be treated in cases of moderate-to-severe skin laxity.

## Conclusions

The injectable collagen biostimulator PLLA-SCA offers a versatile, non-invasive, aesthetic treatment option for specific body areas to address age-associated changes in volume and skin appearance. The evidence base currently consists largely of open-label, prospective studies and case series although systematic evaluations for a number of body areas are in progress. The current data suggest contouring effects and improvements in aesthetic appearance (e.g. wrinkling, dimpling, laxity, cellulite) that are sustained over time with minimal side effects. Standardization of injection protocols for different body areas for different desired outcomes (e.g., improvements in contouring or skin laxity) are now needed in addition to the validation of clinical tools that can be used to agree on goals of treatment and evaluate aesthetic improvements over time.
